# First Insights into the Microbiome of a Mangrove Tree Reveal Significant Differences in Taxonomic and Functional Composition among Plant and Soil Compartments

**DOI:** 10.3390/microorganisms7120585

**Published:** 2019-11-20

**Authors:** Witoon Purahong, Dolaya Sadubsarn, Benjawan Tanunchai, Sara Fareed Mohamed Wahdan, Chakriya Sansupa, Matthias Noll, Yu-Ting Wu, François Buscot

**Affiliations:** 1 Department of Soil Ecology, UFZ-Helmholtz Centre for Environmental Research, 06120 Halle (Saale), Germany; dolaya.sadubsarn@hs-furtwangen.de (D.S.); tanunchai.benjawan@ufz.de (B.T.); sara-fareed-mohamed.wahdan@ufz.de (S.F.M.W.); chakriya.sansupa@gmail.com (C.S.); francois.buscot@ufz.de (F.B.); 2 Department of Bio and Process Engineering, Faculty of Medical Life and Science, Furtwangen University, 78054 VS-Schwenningen, Germany; 3 Department of Botany, Faculty of Science, Suez Canal University, 41522 Ismailia, Egypt; 4 Biology Department, Faculty of Science, Chiang Mai University, Chiang Mai 50200, Thailand; 5 Graduate School, Chiang Mai University, Chiang Mai 50200, Thailand; 6 Institute for Bioanalysis, Coburg University of Applied Sciences and Arts, 96450 Coburg, Germany; matthias.noll@hs-coburg.de; 7 Department of Forestry, National Pingtung University of Science and Technology, Pingtung 91201, Taiwan; 8 German Centre for Integrative Biodiversity Research (iDiv), 04103 Leipzig, Germany

**Keywords:** bacterial diversity, fungal diversity, endophytic phytopathogens, fungal cenoses, halophytic plants, mangrove forest ecosystem, next-generation sequencing, plant epigeous compartments, *Rhizophora stylosa* microbiome

## Abstract

Mangrove forest trees play important ecological functions at the interface between terrestrial and marine ecosystems. However, despite playing crucial roles in plant health and productivity, there is little information on microbiomes of the tree species in mangrove ecosystems. Thus, in this study we aimed to characterize the microbiome in soil (rhizosphere) and plant (root, stem, and leaf endosphere) compartments of the widely distributed mangrove tree *Rhizophora stylosa*. Surprisingly, bacterial operational taxonomic units (OTUs) were only confidently detected in rhizosphere soil, while fungal OTUs were detected in all soil and plant compartments. The major detected bacterial phyla were affiliated to Proteobacteria, Actinobacteria, Planctomycetes, and Chloroflexi. Several nitrogen-fixing bacterial OTUs were detected, and the presence of nitrogen-fixing bacteria was confirmed by *nifH* gene based-PCR in all rhizosphere soil samples, indicating their involvement in N acquisition in the focal mangrove ecosystem. We detected taxonomically (54 families, 83 genera) and functionally diverse fungi in the *R. stylosa* mycobiome. Ascomycota (mainly Dothideomycetes, Eurotiomycetes, Sordariomycetes) were most diverse in the mycobiome, accounting for 86% of total detected fungal OTUs. We found significant differences in fungal taxonomic and functional community composition among the soil and plant compartments. We also detected significant differences in fungal OTU richness (*p* < 0.002) and community composition (*p* < 0.001) among plant compartments. The results provide the first information on the microbiome of rhizosphere soil to leaf compartments of mangrove trees and associated indications of ecological functions in mangrove ecosystems.

## 1. Introduction

Mangrove forest ecosystems encompass genetically diverse communities at interfaces between terrestrial and marine ecosystems in many subtropical and tropical regions [1]. These transition ecosystems provide spawning and breeding grounds, nurseries, and habitats for many aquatic, semi-aquatic and terrestrial animals. Root systems of mangrove trees also provide natural barriers that reduce impacts of storms and protect coasts from erosion [2]. Moreover, mangrove forests are not only ecologically important but also economically valuable [2,3]. Many mangrove tree species are used for timber, fuelwood, and charcoal [2]. Mangrove forests are also rich sources of honey, and diverse medicinal, cosmetic, and other products [2,3]. In addition, they now attract many eco-tourists, anglers, and birdwatchers, thus contributing to incomes of countries with coastal mangroves [2,3]

Plants′ microbiomes play crucial roles in their health and productivity [4]. Many researchers have successfully applied knowledge acquired about plant microbiomes to produce specific inocula for crop protection [5,6]. Such inocula can stimulate plant growth by releasing phytohormones and enhancing uptake of some mineral nutrients (particularly phosphorus and nitrogen) [6,7,8]. However, most of the plant microbiome studies have focused on the model plant *Arabidopsis thaliana* and economically important crop plants, such as barley (*Hordeum vulgare*), corn (*Zea mays*), soybean (*Glycine max*), rice (*Oryza sativa*), and wheat (*Triticum aestivum*), but there is less information on microbiomes of tree species [4,6]. Plant microbiomes are determined by plant-related factors (e.g., genotype, organ, species, and health status) and environmental factors (e.g., land use, climate, and nutrient availability) [4,8]. Two of the plant-related factors, plant species and genotypes, have been shown to play significant roles in shaping rhizosphere and plant microbiomes, as tree genotypes and species are associated with specific microbial communities [7]. Different plant organs also have specific microbial communities depending on plant-associated factors (plant genotype, available nutrients, and organ-specific physicochemical conditions) and/or environmental conditions (associated with aboveground and underground surfaces and disturbances) [9,10,11].

However, there is very limited information on microbiomes of tree species growing in mangrove forests, and most studies that have addressed them have focused on specific compartments of mangrove trees, such as rhizosphere, roots, or leaves [12,13]. Few have considered more than two compartments associated with different plant organs [10]. Thus, the inter-relationships of microbial communities in different plant compartments are still poorly understood.

To assist efforts to improve understanding, in this study we aimed to characterize the microbiome of the mangrove tree species *Rhizophora stylosa*, one of the most dominant species in widely distributed brackish environments across Asia and Australia [14]. The studied microbiome compartments included root, stem, and leaf endospheres, and the soil rhizosphere. We tested and/or quantified effects of soil and plant compartments (and selected physicochemical properties of plant compartments) on the richness and composition of bacterial and fungal communities, as well as the proportions (and taxonomic affiliations) of microbes capable of colonizing at least two compartments. We also explored potential ecological functions of microbes in the rhizosphere. We hypothesized that different compartments significantly differ in richness and composition of bacterial and fungal communities, and that these differences are related to differences in the compartments′ physicochemical properties. For example, in mangrove ecosystems, salinity is high in rhizosphere soil and roots, but low in stems and leaves. We expected to detect differences in macronutrient contents among the compartments that affect the community composition of their microbiomes. We also hypothesized that some microbes can colonize more than one compartment, and some may even be detectable in all compartments from the rhizosphere to leaf. Such microbes must be adapted to both high and low salinity. Potential soil microbial ecological functions were characterized by measuring activities of important soil enzymes for the acquisition of macronutrients such as carbon (C; β-glucosidase), nitrogen (N; N-acetylglucosaminidase), and phosphorus (P; acid phosphatase) [15].

## 2. Materials and Methods

### 2.1. Study Site, Experimental Design, and Sample Processing

We selected five healthy, mature *R. stylosa* trees for sampling in spring (March) 2018. The trees were 170–200 cm tall, and growing in Zuo-An (Right Bank) wetland, located near a wastewater area in Pingtung County (22°26′17.6″ N 120°29′29.6″ E), southern Taiwan. Pingtung County has a tropical climate with an average annual rainfall of 1000 mm in coastal regions where the samples were taken, with minimum and maximum monthly temperature ranging from 14 to 25 °C in January and 25 to 33 °C in July [16]. Dominant mangrove trees in the Zuo-An wetland are *R. stylosa*, *Kandelia candel,* and *Avicennia marina*. Four compartments of the five selected trees and associated soil (root, stem and leaf endospheres and rhizosphere soil) were sampled, targeting two phylogenetic groups: bacteria and fungi. We sampled the stem wood using a Makita BDF 451 cordless drill equipped with a wood auger (diameter, 20 mm), which was dipped into alcohol, flamed, and wiped with ethanol between drillings to avoid cross-contamination. The bark was completely removed from each selected part of a stem using a sterile knife before drilling. The drill was operated slowly and introduced perpendicularly to the stem axis at two positions (50 and 100 cm from the ground). The two stem samples were pooled to make a composite sample per tree. Eight leaves from three positions (top, middle, and bottom branches) per tree were also pooled to make a composite sample. In addition, four stilt root samples with rhizosphere soil from four directions around each tree were collected and pooled to make a composite sample. All samples were transported on ice to laboratory. Rhizosphere soil was removed by washing roots with molecular biology grade DNA-, RNAse-, and DNAse-free water (AppliChem, Darmstadt, Germany). Roots and leaves were then washed in Milli-Q water five times, 70% ethanol (for 7 min) and Milli-Q water three times then placed in Milli-Q water for 1 h at room temperature. Each surface-sterilized root sample was subsampled, cut into small pieces (<2 mm long) using sterile scissors and then mixed with each other. Each surface-sterilized leaf sample was cut into small pieces (<1 cm^2^) using sterile scissors and ground into a fine powder using liquid nitrogen and a swing mill (Retsch, Haan, Germany).

### 2.2. Soil Physicochemical Parameters

Total carbon (C) and nitrogen (N) concentrations in rhizosphere soil and plant compartments were measured by dry combustion at 1000 °C with a CHNS-Elemental Analyzer (Elementar Analysensysteme GmbH, Hanau, Germany). The pH and electric conductivity of rhizosphere soil samples were determined using a WTW Multi 3510 IDS portable meter (Weilheim, Germany). Cation (K, Mg, Ca, and Na) contents in plant compartments were determined by atomic absorption spectrophotometry, using a Z 5300 instrument following recommendations of the manufacturer (Hitachi—Science & Technology, Tokyo, Japan).

### 2.3. Analysis of Soil Rhizosphere Enzyme Activities

To explore potential ecological functions in the rhizosphere of each individual tree, we analyzed activities of three important enzymes for C, N, and P acquisition—β-glucosidase (EC 3.2.1.21), N-acetylglucosaminidase (EC 3.1.6.1), and acid phosphatase (EC 3.1.3.2), respectively—in the soil samples, using 4-methylumbelliferone (MUB) derivatives and previously described methodology [15]. To prepare soil slurries, 50 mL of 50 mM sodium acetate buffer (pH 5.5) were added to 0.5 g portions of soil (wet weight, equivalent to 0.098–0.183 g dry weight) and homogenized for 5 min in a bath sonicator. 4-MUB-β-glucopyranoside, 4-MUB-phosphate, and 4-MUB-N-acetyl-β-glucosaminide (150, 150 and 200 μmol/L) substrate solutions were prepared for the β-glucosidase, acid phosphatase, and N-acetylglucosaminidase assays, respectively. Substrate blank wells were filled with 50 μL substrate solution and 200 μL acetate buffer. Assay wells were filled with 50 μL substrate solution and 200 μL soil slurry. Quench coefficient and emission coefficient wells were filled with 50 μL of a MUB dilution series (2.5, 1.25, 0.625, 0.16 μmol/L) and 200 μL soil slurry or 200 μL acetate buffer. To prepare homogenized blanks, 200 μL soil slurry and 50 μL acetate buffer were mixed. Plate blanks were prepared by only adding 250 μL acetate buffer. There were eight replicate wells for each substrate blank, assay, quench standard, emission standard, homogenate blank, and plate blank. The microplates were incubated in the dark at room temperature for 60 min. To stop the reactions, 10 μL of 1.0 M NaOH was added to each well approximately one minute before reading the plates. A FLUOstar OPTIMA microplate reader (BMG Labtech, Ortenberg, Germany) with 355 nm excitation and 460 nm emission filters was then used for measuring the fluorescence intensities of each well. After correcting for blanks, emission, and quenching, activities of the three enzymes were expressed in units of nmol h^−1^ g dry soil^−1^.

### 2.4. Microbiome Analysis

DNA was extracted from approximately 150 mg of each homogenized soil sample using a ZR Soil Microbe DNA MiniPrep kit (Zymo Research, Irvine, CA, USA) according to the manufacturer′s protocol. The presence and quantity of genomic DNA were checked using a NanoDrop ND-1000 spectrophotometer (ThermoFisher Scientific, Dreieich, Germany). DNA extracts were then stored at −20 °C for further analysis. Bacterial and fungal amplicon libraries were obtained separately for Illumina sequencing using the primer combination 341F (5′CCTACGGGNGGCWGCAG3′) [17] and 785R (5′GACTACHVGGGTATCTAAKCC3′) [17], which target the V3–V4 region of the 16S rRNA gene, as well as fITS7 (5′GTGARTCATCGAATCTTTG3′) [18] and ITS4 (5′TCCTCCGCTTATTGATATGC3′) [19], which target the fungal ITS2 region. PCR conditions are shown in the Supplementary Materials. Amplification products were visualized with eGels (Life Technologies, Grand Island, NY, USA). Products of three replicate reactions per sample were then pooled in equimolar amounts, and each pool was cleaned with a Diffinity RapidTip (Diffinity Genomics, West Henrietta, NY, USA), then sequenced using an Illumina MiSeq platform and V3 Chemistry (Illumina) by RTL Genomics (Lubbock, TX, USA). The presence of nitrogen-fixing bacteria in samples was verified by PCR using *nifH* gene-specific primers (PolF/PolR), as previously described [20].

### 2.5. Bioinformatic Analysis

The quality of reads from the paired-end sequences generated by the Illumina MiSeq sequencing platform was first examined using MOTHUR [21] and OBI tools software [22]. Reads fulfilling the following criteria were remained for further analyses: A minimum average quality of 25 Phred score; containing homopolymers with a maximum length of 20 nucleotides without ambiguous nucleotides. We detected chimeric sequences using the UCHIME algorithm, as implemented in MOTHUR and removed them from the datasets. The obtained reads were then clustered into operational taxonomic units (OTUs) using the CD-HIT-EST algorithm with a threshold of 97% sequence similarity [23]. The OTU representative sequences (defined as the most abundant sequence in each OTU) were taxonomically assigned by alignment with reference sequences in the SILVA 132 database for the bacterial 16S rRNA gene [24] and UNITE database (version unite.v7) [25] for fungal ITS, using the naive Bayesian classifier as implemented in MOTHUR with default parameters. They were also assigned to ecologically functional groups of bacteria and fungi using Functional Annotation of Prokaryotic Taxa (FAPROTAX) [26] and FUNGUILD [27], respectively. Rare OTUs (singletons to tripletons), which could potentially originate from artificial sequences, were removed. The read counts were normalized with respect to the smallest read number per sample (as detailed in the Results section). The raw sequence datasets are available in the National Center for Biotechnology Information database (NCBI) under the BioProject ID PRJNA554586.

### 2.6. Statistical Analysis

The acquired data were analyzed using PAST software version 2.17c [28]. Measures of bacterial and fungal OTU richness, including Shannon diversity indices, were calculated using the PAST function “diversity indices”. To assess the coverage (sequencing depth), data pertaining to each sample was subjected to rarefaction analysis using the PAST function “diversity” (Figure S1). Bacteria were only confidently detected in the rhizosphere compartment; thus, we did not perform further statistical analyses of effects of soil and plant compartments on their richness and community composition. Differences in fungal richness among the soil and plant compartments were analyzed using the Kruskal-Wallis (KW) test as their variances were not sufficiently homogenous for One-way ANOVA according to Levene′s test. Data on the abundance of OTUs in the microbiome communities were converted to presence/absence data, as relative abundances derived from next-generation sequencing may be biased and should not be used for absolute quantification [29]. However, abundance data (numbers of sequencing reads) were used for heatmapping N-fixing bacteria in rhizosphere soil samples. Percent occurrence data were used for heatmapping dominant fungi in all soil-plant compartments (≥80% in at least one compartment). Non-metric multidimensional scaling (NMDS) ordination based on presence/absence data and Jaccard distances in R (version 3.5.3) was used to investigate and visualize the composition of fungal communities in different soil and plant compartments. The Envfit function of the Vegan package in R was also used to investigate correlations between factors (C, N, K, Mg, Ca, Na) and the composition of the fungal communities (Table 1). The bacterial community in rhizosphere soil was subjected to corresponding NMDS and Envfit analyses. One-way analysis of similarities (ANOSIM) and nonparametric multivariate analysis of variance (NPMANOVA) were used to test the differences in fungal community composition among the soil and plant compartments using presence/absence data and Jaccard distances (Table 2). Significance levels were based on 999 permutations. As more than two groups were compared, Bonferroni-corrected *p* values were applied. ANOSIM yields a sample statistic (*R*) indicating the degree of separation between test groups, with values ranging from −1 to 1 (*R* = 0–0.24, no separation to barely separated; *R* = 0.25–0.75, separation with different degrees of overlap; *R* > 0.75–1, well separated) [30]. Nutrients and their associated parameters were compared among the plant compartments using ANOVA. All datasets were tested for normality using the Jarque-Bera test and equality of variance using Levene′s test. Data are presented as means and standard errors unless otherwise stated.

## 3. Results

### 3.1. General Traits of the Microbiome Associated with the Mangrove Tree Rhizophora Stylosa

**Figure 1:**
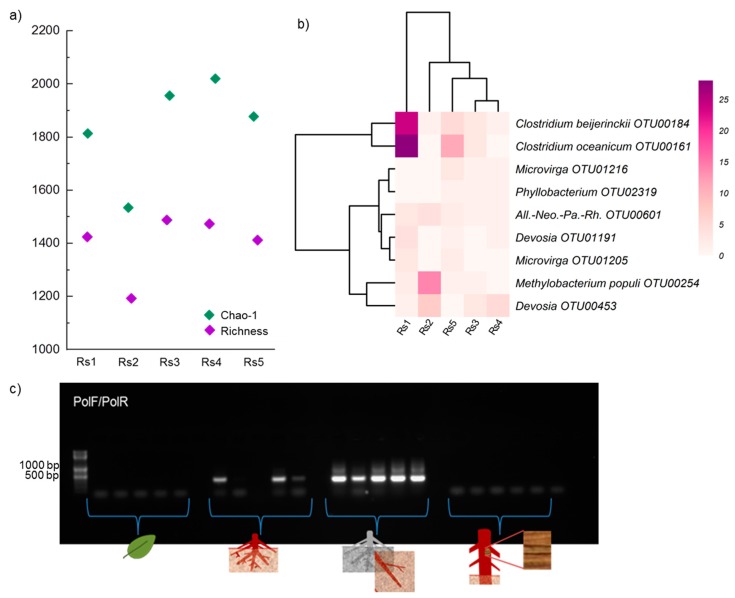
(**a**) Observed and Chao1-estimated richness of the bacterial microbiome in the five samples of rhizosphere soil (Rs1-Rs5), (**b**) heatmap of N-fixing bacteria (based on abundance data) in the samples, and (**c**) PCR results obtained using PolF/PolR primers to detect the *nifH* gene (360-bp) in the four soil and plant compartments (left to right: leaf, root, rhizosphere soil, and stem).

Surprisingly, bacteria were only detected in all rhizosphere soil samples and only a few root endosphere samples. Despite testing various bacterial primer sets, only weak signals or no bacterial PCR products were obtained from root, stem, and leaf samples (Figure S2). Therefore, only rhizosphere soil samples were subjected to bacterial sequencing. In total, 66,263 sequences of bacterial 16S rRNA gene were detected in the rhizosphere soil samples, on average 13,252 ± 187 per sample, after removing non-target and chimeric sequences. Singletons to tripletons were removed and the sequences were subsampled and rarefied to the smallest read numbers per sample (7086). Finally, 2497 different bacterial OTUs were obtained and used for further analyses. All the rarefaction curves indicated that the sampling effort was sufficient to capture each sample′s overall composition (Figure S1). On average, OTU richness and chao-1 estimated richness values of 1394 ± 53 and 1837 ± 84 were obtained for the five rhizosphere soil samples (Figure 1).

Fungi were detected in all soil and plant compartments. In total, 278,409 quality-filtered fungal ITS sequences were obtained from rhizosphere soil and plant compartment samples (13,920 ± 1734 on average per sample) after removing non-target and chimeric sequences. Singletons to tripletons were removed and the sequences were subsampled and rarefied to the smallest read numbers per sample (7542). Finally, 455 fungal OTUs were obtained and used for further analyses. All the rarefaction curves indicated that the sampling effort was sufficient to capture each sample′s overall composition (Figure S1).

### 3.2. Bacterial Community in Rhizosphere Soil

**Figure 2:**
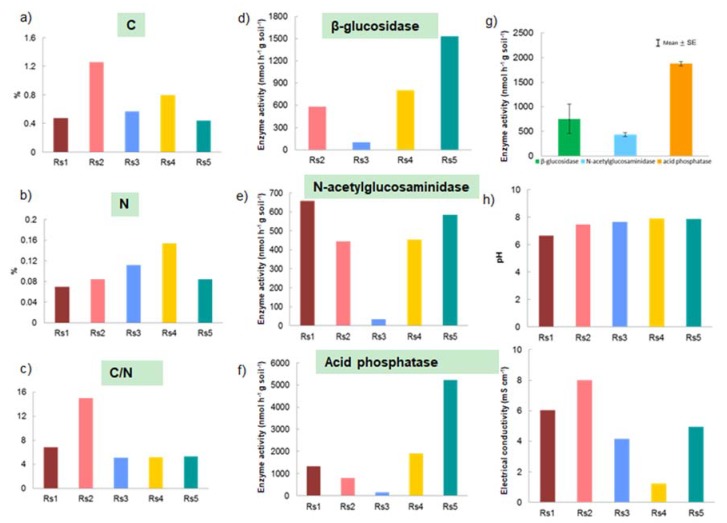
(**a**–**c**) Carbon content (C), nitrogen content (N) and carbon/nitrogen ratio (C/N); (**d**–**f**) β-glucosidase, N-acetylglucosaminidase and acid phosphatase enzyme activities; (**g**) mean activity of β-glucosidase, N-acetylglucosaminidase and acid phosphatase; (**h**) pH; and (**i**) electrical conductivities in the five rhizosphere soil samples (Rs1–Rs5).

Proteobacteria (856 OTUs), Actinobacteria (342 OTUs), Planctomycetes (358 OTUs), Chloroflexi (233 OTUs), and Firmicutes (161 OTUs) were the main bacterial taxa detected in the sampled soil of the *R. stylosa* rhizosphere (for details, see Supplementary Table S1). We successfully assigned 396/2497 bacterial OTUs into about 30 ecological functional groups (Figure S3). The frequently detected ecological functional groups (>2% of total functionally assigned OTUs) were aerobic chemoheterotrophy, sulfate respiration, fermentation, cyanobacteria, intracellular parasites, predatory, aromatic compound degradation, and nitrogen fixation (Supplementary Table S1, Figure S3). Sulfate respiration (122 OTUs, 31% of total functionally assigned OTUs) was mostly affiliated to Deltaproteobacteria (Desulfobulbaceae, Desulfobacteraceae, Desulfarculaceae, Syntrophaceae, Desulfohalobiaceae, Desulfuromonadaceae, and Desulfovibrionaceae). Nitrogen fixation was affiliated to nitrogen-fixing bacteria, like *Allorhizobium-Neorhizobium-Pararhizobium-Rhizobium*, *Methylobacterium* (*Methylobacterium populi*), *Devosia*, *Microvirga*, *Phyllobacterium,* and *Clostridium* (Figure 1). The presence of nitrogen-fixing bacteria was checked by *nifH* gene-based PCR using PolF/PolR primers (Figure 1c). The rhizosphere soil bacterial community was marginally significantly correlated with the C/N ratio, but not other physicochemical properties (Figure 2, Supplementary Table S2).

### 3.3. Differences in Fungal Phylogenetic and Functional Community Composition among Soil and Plant Compartments

**Figure 3:**
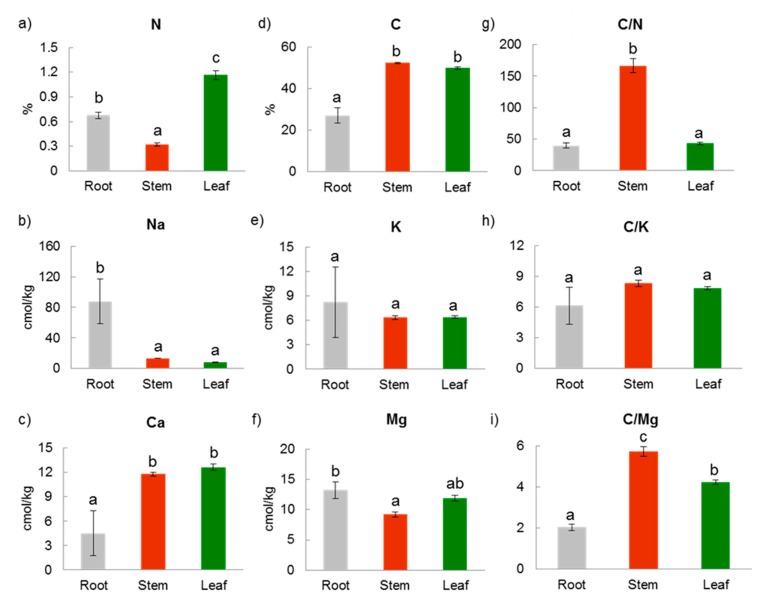
Measured values in root, stem, and leaf compartments of: (**a**) nitrogen (N), (**b**) sodium (Na), (**c**) calcium (Ca), (**d**) carbon (C), (**e**) potassium (K), (**f**) magnesium (Mg) contents, (**g**) carbon/nitrogen (C/N) ratio, (**h**) carbon/potassium (C/K) ratio, and (**i**) carbon/magnesium (C/Mg) ratio (means ± SE). Different letters above bars within panels indicate significant differences (*p* < 0.05) according to one-way analysis of variance (ANOVA).

Plant compartments differed in physicochemical properties, including carbon (C) and nitrogen (N) contents, concentrations of major cationic nutrients (K, Mg, Ca), and salinity (Na) (Figure 3, Supplementary Table S3). The leaf plant compartment had high N, C, Ca, and Mg contents. Stems and leaves had similar, high C content, Ca concentration, C/K and C/Mg ratios, but low Na concentration (Figure 3). The stem compartment had the highest C/N and C/Mg ratios (Figure 3). Roots had higher concentrations of Na and Mg. Rhizosphere had much lower concentrations of both C and N than the other plant compartments (Figure 2 and Figure 3).

**Figure 4:**
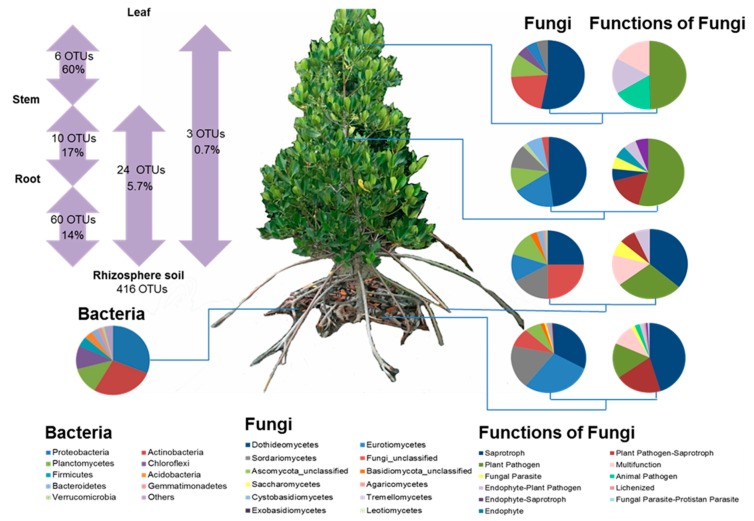
Bacterial taxonomic community composition in the rhizosphere soil and fungal taxonomic community composition in all four rhizosphere soil and plant compartments. Information on the fungal ecological functional groups is also provided. Proportions of fungal OTUs that can colonize at least two of the compartments are shown in the left panel.

Fungal communities differed between plant compartments (Figure 4). The major groups of fungi were Dothideomycetes (146 OTUs; including *Cladosporium*, *Toxicocladosporium strelitziae*, *Hortaea werneckii*, *Phaeophleospora hymenocallidicola*), Eurotiomycetes (125 OTUs; including *Penicillium coffeae*, *Penicillium citrinum*), and Sordariomycetes (76 OTUs; including *Xylariaceae*, *Bartaliniaceae*, *Monosporascus*). Dothideomycetes had the highest relative sequence abundance in every compartment and was dominant in stem and leaf compartments (accounting for ≥40% of total detected OTUs in them). In contrast, diversity of Eurotiomycetes was highest in the rhizosphere compartment (121 OTUs), but lowest in root (9 OTUs) and leaf (1 OTU) compartments. Similarly, the diversity of Sordariomycetes was highest in rhizosphere soil (68 OTUs), followed by root (12 OTUs), stem (7 OTUs), and leaf (1 OTU) compartments.

Affiliated functional guilds also varied across the compartments (Figure 4). We successfully assigned 134/455 fungal OTUs into about 11 functional guilds (Figure 4). Plant pathogens were the most abundant functional group detected in all compartments, but were particularly more frequently detected in stems and leaves (accounting for ≥50% of total functionally assigned OTUs detected in stem and leaf samples). Most OTUs in rhizosphere and root compartment corresponded to saprotrophs (≥35% of total functionally assigned OTUs detected in rhizosphere soil and root samples), which were barely detected in the stem samples and not detected at all in the leaf compartment. Plant pathogen-saprotrophs were moderately abundant in the rhizosphere soil and stem compartments (accounting for ≥15% of total functionally assigned OTUs detected in them), but were not detected in the leaf compartment. In turn, endophyte-plant pathogens were frequently detected in the leaf compartment (17% of total functionally assigned OTUs detected in leaf) but less frequently in the stem, root, and rhizosphere compartment (2 to 7% of total functionally assigned OTUs) (see Figure 4).

### 3.4. Effects of Plant Compartments on Microbial Richness

**Figure 5:**
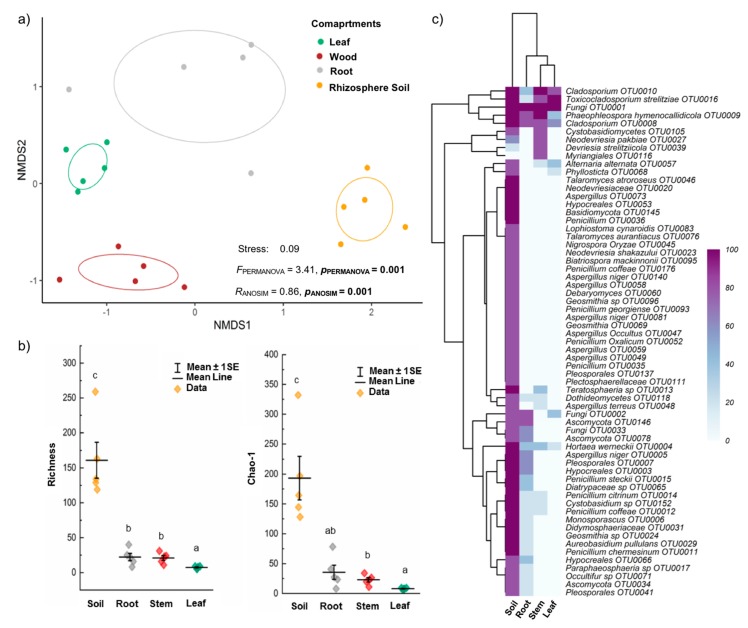
Fungal community composition and richness across the *R. stylosa* (rhizosphere soil and plant compartments: (**a**) non-metric multidimensional scaling plot of fungal communities in indicated compartments, showing 95% confidence ellipses around mean values of NMDS axes 1 and 2; (**b**) fungal OTU and Chao1-estimated OTU richness (means ± SE), and (**c**) heatmap of dominant fungal OTUs based on percent occurrence data (accounting for ≥ 80% of OTUs in at least one compartment). Different letters above OTU/Chao1 richness bars within a panel indicate significant differences (*p* < 0.05) according to analysis of variance or Kruskal-Wallis tests.

Fungal OTU richness was significantly affected (*H* = 15.07, *p* = 0.0017) by plant compartments (Figure 5). Rhizosphere soil samples had the highest richness (416 OTUs in total, 160.8 ± 25.6 on average), while leaves had the lowest richness (19 OTUs in total, 7.4 ± 0.8 on average). Fungal OTU richness in the root compartment (19 OTUs in total, 22.2 ± 5.3 on average) and stem compartment (61 OTUs in total, 21.0 ± 3.5 on average) were not significantly different. Observed and Chao1-estimated richness vales were consistent (Figure 5).

### 3.5. Effects of Plant Compartments and Their Physicochemical Properties on Fungal Community Composition

Fungal communities significantly differed among plant compartments (*F*_PERMANOVA_ = 3.41, *p* = 0.001, *R*_ANOSIM_ = 0.86, *p* = 0.001), and NMDS ordination clearly separated them in terms of community composition (Figure 5). In addition, Na, Ca, and C contents and C/N ratio significantly correlated with the plant compartments′ fungal community composition (Table 1), and both C content and C/N ratio significantly correlated with the composition of the fungal communities of all soil and plant compartments (Supplementary Table S4).

### 3.6. Microbes Capable of Colonizing At Least Two of the Soil-Plant Compartments

A distinct fungal community composition was associated with each plant compartment (Figure 4). Proportions of the fungal class Dothideomycetes at OTU level were very high in leaf samples and gradually decreased in stem, root, and rhizosphere soil samples. In contrast, proportions of Eurotiomycetes were high in rhizosphere soil and gradually decreased in root, stem, and leaf samples. Proportions of the fungal class Agaricomycetes decreased from root through stem to leaf compartments. Two fungal classes (Tremellomycetes and Saccharomycetes) were detected only in the rhizosphere soil compartment.

We analyzed distributions of each OTU that could colonize at least two compartments of rhizosphere soil and the internal plant tissues upwards from roots to leaves (and vice versa from leaves to rhizosphere soil) (Figure 4). Sixty fungal OTUs detected in rhizosphere soil (14% of the total) were also detected in the root endosphere and thus could have been transported between these two compartments. Similarly, 17% of fungi detected in the root endosphere were also found in the stem compartment (10 OTUs), and 10% of the fungi detected in the stem were also detected in the leaf endosphere compartment (six OTUs). In addition, 5.7% of rhizosphere soil fungi were detected in the stem, but not the root compartment (24 OTUs). Only 0.7% of the rhizosphere soil fungi were found in the leaves (three OTUs), but not the root and stem compartments. Overall, only six fungal OTUs (mainly plant pathogens) were detected in all soil-plant compartments. One of these (Otu0001) was an unclassified fungus, while the other five were assigned to the Dothideomycetes, and defined as *Phaeophleospora hymenocallidicola* (Otu0009, SH1209921.08FU), *Hortaea werneckii* (Otu0004, SH1154533.08FU), *Toxicocladosporium strelitziae* (Otu0016, SH1190879.08FU), and twice *Cladosporium* (Otu0008, Otu0010). Both *Cladosporium* OTUs were classified according to UNITE Species hypotheses as *Mycosphaerella tassiana* (SH1190878.08FU, current name *Cladosporium herbarum*).

### 3.7. Potential Soil Microbial Ecological Functions

Activities of the important enzymes for C, N, and P acquisition differed in the five rhizosphere soil (Rs) samples, declining in the order Rs5 > Rs4 > Rs1 > Rs2 > Rs3 (Figure 2). Acid phosphatase and β-glucosidase activities were highest in sample Rs5, while Rs3 had the lowest activities of these two enzymes (Figure 2). NAG activity was similar in all samples, except Rs3, which had the lowest activity. We investigated the rhizosphere microbial communities associated with Rs5 and Rs3 samples (which had the highest and lowest enzyme activities, respectively) and linked them with potential enzyme activities. Relative abundances of abundant bacteria were similar in Rs5 and Rs3 samples (Supplementary Table S5), but Rs5 had higher abundance of various fungi, including *Aspergillus* spp. (*A. niger* and *A. flocculosus*) and *Penicillium* spp. (*P. steckii*, *P. coffeae* and *P. citrinum*) than sample Rs3, especially *A. niger* (550 sequence reads in Rs5 vs. 88 in Rs3) (Supplementary Table S6).

## 4. Discussion

We provide the first information on mangrove tree microbiomes, encompassing root, stem and leaf endospheres and the rhizosphere. Furthermore, by showing that some fungi can colonize different plant compartments we contribute to disentangling their propagation channels within plants. Our results also reveal that fungi dominate the plant endosphere microbiome of healthy *R. stylosa*, which seems to be almost devoid of bacteria, at least at our study site. In this section, we compare and discuss the microbiome of *R. stylosa* and other model plants.

### 4.1. Fungi Dominate Internal Microbiomes of R. stylosa

Bacteria and fungi were both present in the rhizosphere, but only fungi were confidently detected in the internal plant compartments. It should be noted that although they are sometimes referred to as a subset of the rhizosphere microbiome, endophytic bacteria can greatly differ from rhizospheric bacteria [31], and many studies have shown that the majority of rhizospheric bacteria cannot invade intact plant tissues [31,32,33]. Accordingly, we only detected a few endogenous bacteria in the roots, and most rhizospheric bacteria were not detected in any endospheric compartment of *R. stylosa*. To exclude the possibility that groups of bacteria other than those of the rhizosphere might colonize several endospheric compartments, we tested several general bacterial primer pairs to detect endospheric bacteria of *R. stylosa*. However, we only obtained weak signals of PCR products of sequences associated with the 16S rRNA gene in some root, stem and leaf endosphere samples, suggesting that these plant compartments have very low bacterial biomass. The low bacterial biomass in leaves and other plant organs may be related to antibacterial agents produced by the plants [34]. Sources of such weak signals in the root endosphere may be nitrogen-fixing bacteria, as we detected the *nifH* gene in some DNA extracts of the root samples (Figure 1). Overall, our data suggest that fungi dominate the microbiomes in internal compartments of *R. stylosa*.

We found significant differences among internal compartments of *R. stylosa* in fungal community composition and richness, possibly linked to plant-associated and environmental factors [9,35]. Similar patterns have also been observed in other terrestrial and riparian woody plants in which the endospheric mycobiome was found to be compartment-specific [35,36]. In our study, the leaf endosphere compartment had lower richness of fungal OTUs (~8 OTUs) than the other compartments. It should be noted that in a study of leaf-inhabiting fungi of *R. stylosa* in south China, Yao et al. (2019) found on average 57 OTUs, much more than we detected. This may reflect the strength of effects of location and environmental factors shaping fungal richness.

### 4.2. Ascomycota Was the Most Frequently Detected Fungal Phylum

The main fungal classes were Dothideomycetes, Eurotiomycetes, and Sordariomycetes—members of the Ascomycota that were detected in a previous study of epiphytic and endophytic fungi of mangrove tree species’ leaves [13]. However, substantial proportions of fungi of the phylum Basidiomycota (Tremellomycetes, Microbotryomycetes, Exobasidiomycetes) in the endospheric leaf compartment of *R. stylosa* (~36%) were detected in the cited study [13]. We found only one OTU of Exobasidiomycetes (*Sympodiomycopsis paphiopedili*) and no members of the Tremellomycetes or Microbotryomycetes in the leaf endosphere compartment of *R. stylosa*, although the primers used were capable of amplifying such Basidiomycota sequence targets. Tremellomycetes were exclusively detected in rhizosphere soil. Other classes of Basidiomycota detected in our study are Cystobasidiomycetes and Agaricomycetes, which have also been detected in some previous studies of mangrove ecosystems [13,37].

### 4.3. Factors Responsible for the Microbiome Speciation among Compartments of the Investigated Soil-Plant System

The differentiation of plant microbiomes in different soil-plant compartments may be driven by niche-based processes (which require variation in local environmental conditions) [10], especially in cases such as this, where all the selected *R. stylosa* trees were located in the same ecosystem with less than 1 km between them and no visible dispersal barriers. There may be several reasons for the differences in microbiomes detected among the soil and plant compartments in our study [10]. First, there may be differences in local species pools that colonize each compartment, for example, the fresh and marine water and soil microbial pool for roots, and airborne and rainfall microbial pools for stems and leaves. Air- and rain-borne microbial pools can reach soil and potentially enter roots and other plant compartments, but in those compartments, they have to compete with existing soil microbial communities and be able to colonize the endosphere of root tissue. Second, there may be influential local biotic factors, for example, plants may promote establishment of beneficial, mutualistic microbes by secreting chemicals from specific organs that attract them. Third, abiotic factors may also be important, as different plant (and soil) compartments have different physicochemical properties (Figure 2 and Figure 3). We found, for example, significant differences among plant organs in C content, C/N ratio and Ca concentration that significantly correlated with their fungal community composition. The composition of the bacterial community at rhizosphere level also marginally correlated with C/N values. These macronutrients (C, N and Ca) are highly relevant for microbial activity and growth [38,39]. C and N contents are sum parameters for cell activities [38,39]. Ca^2+^ is necessary for maintenance of cell structure, motility, and cell division, and affects the permeability of ions, sugar, and amino acids [40,41]. Thus, these macronutrients determine or partly shape the microbiome structure.

### 4.4. Microbes in Extreme Environments: Na as a Factor Correlating with Microbial Community Composition

Mangrove soils are typically saline, with moderate to high levels of Na, which can also be transported to plant compartments especially by roots. Thus, the Na concentration was expected to be significantly correlated to microbial community composition. We confirmed this hypothesis, as we detected significant correlations between Na and fungal community composition. Electrical conductivity measurements of the rhizosphere soil compartment showed that it was mostly moderately saline. Moreover, high frequencies of halotolerant and halophilic microbes, such as Proteobacteria (genera *Halomonas* and *Altererythrobacter*), Actinobacteria (*Isoptericola* *halotolerans*), Firmicutes (*Halobacillus trueperi* and *Bacillus* *jeotgali*), and Bacteroidetes (*Muricauda aquimarina*) were found in the rhizosphere compartment. Many of these bacteria have been previously detected in saline soils [42]. Our sampling area is a wastewater area and we also frequently detected Caldilineaceae and sulfate reducing bacteria (Desulfobulbaceae, Desulfobacteraceae, Desulfarculaceae, Syntrophaceae, Desulfohalobiaceae, Desulfuromonadaceae, and Desulfovibrionaceae) (Supplementary Table S1 and Figure S3). Some studies have found some of these bacterial families in typical municipal wastewater treatment plants [43]. We also found halotolerant fungi, especially the yeast *H.* *werneckii*, a dominant black yeast species that can survive in hypersaline waters [44,45,46]. *A. niger*, which can also survive in hypersaline environments [47], was also frequently detected.

### 4.5. Potential Soil Microbial Ecological Functions

We detected considerable variation in activities of important enzymes for C, N, and P acquisition (especially acid phosphatase and β-glucosidase) among the samples of rhizospheric soil associated with different individuals of *R. stylosa*. We found higher relative sequence abundances of *Aspergillus* spp. (especially *A. niger*) and *Penicillium* spp. in samples with high acid phosphatase and β-glucosidase. These taxa might therefore contribute to production of these two enzymes. Previous studies have shown that *A. niger* can produce high amounts of both acid phosphatase and β-glucosidase [48,49]. Furthermore, *Penicillium citrinum* can reportedly produce high amounts of β-glucosidase [50]. Another interesting enzyme to test in the future is xylanase, which plays an important role in decomposition of hemicellulose [51]. This enzyme is reportedly produced by both bacteria (e.g., *Bacillus* spp.) [52] and fungi (*Fusarium* spp. and *Aureobasidium* spp.) [53] in mangrove ecosystems. These potential xylanase-producing microorganisms are detected in our study.

### 4.6. Nitrogen-Fixing Bacteria in Rhizosphere soil

Nitrogen plays important roles in plant growth, development, and health. Bacterial nitrogen fixation can be a significant source of nitrogen in mangrove ecosystems [54,55]. Members of the genera *Rhizobium*, *Phyllobacterium*, and *Clostridium* have been previously identified as nitrogen-fixing bacteria and found in the rhizosphere of mangrove ecosystems [56,57,58,59]. Some of the bacterial 16S rRNA gene sequences we identified were affiliated to known nitrogen-fixing bacteria in the rhizosphere (nine OTUs, representing six genera: *Rhizobium*, *Methylobacterium*, *Devosia*, *Microvirga*, *Phyllobacterium*, *Clostridium*), which were found in all rhizosphere samples (Figure 1). We also detected clear PCR amplicons of the *nifH* gene. Thus, we suggest that nitrogen-fixing bacteria may play an important role in nitrogen acquisition for *R. stylosa*. Interestingly, in this study we did not detect the globally distributed free-living nitrogen-fixing bacteria *Azospirillum* spp., which commonly occur in rhizospheres and roots of land-plants, including mangrove plant species [60,61].

### 4.7. Potential Transport of Pathogens

Only few fungal phytopathogens find success in colonizing all soil-plant compartments, namely those which can be considered general plant pathogens are able to infect different plant districts. As we only investigated such fungi in the internal plant tissues of healthy looking trees (with no visible disease symptoms), the fungi we detected in all soil and plant compartments might be pathogens originating from soil that dwell in all internal plant tissues (root, stem and leaf) during the lifespan of *R. stylosa*. We propose that these plant pathogens could be transported by xylem (Supplementary Figure S4), as reported in other studies [62]. There are limited fungal plant pathogens, which thrive in this nutrient poor environment [62]. In this case, *H. werneckii*, *T. strelitziae*, *P. hymenocallidicola* can survive in this nutrient poor environment. *H. werneckii* is a halotolerant fungus that is involved in the formation of leaf lesions and non-pathological dermal change on human palms called tinea nigra [63]. *T. strelitziae* may cause minute brown lesions on flowers of *Strelitzia reginae* [64], and *P. hymenocallidicola* can cause leaf spot and tip blight disease on spider lily [65]. According to UNITE Species hypotheses *Cladosporium* Otu0008 and Otu0010 were both identified as *M. tassiana* (current name *Cladosporium herbarum*), which was detected in every plant compartment. It is known as a plant pathogen that can cause stigmatomycosis of *Annona muricata* [66]. Overall, our study provided evidence that some diseases associated with aboveground parts of plants in mangrove ecosystems may be caused by pathogens originating from belowground pools.

In summary, we detected few fungi spread over several compartments of *R. stylosa*, and as their frequencies in compartments did not change gradually or systematically, as in environmental gradients, there is low probability that fungi frequently move through the plant compartments from one to another. Furthermore, we find that the communities of each compartment are influenced by their specific physicochemical conditions, supporting the idea that tissues recruit their endogenous mycobiome largely independently.

### 4.8. Comparison of the R. stylosa Microbiome to those of Other Model Plants in Different Ecosystems

*Rhizophora stylosa* is a typical, important tree species of the mangrove ecosystem. Thus, the final goal of our work was to *use R. stylosa* as a model plant to investigate its microbiome and compare it to other model plants in other ecosystems [67], including: the general model plant *Arabidopsis thaliana* [68,69]; the model crop plants *Oryza spp*. (rice) [70,71] and *Zea mays* (maize) [72]; and the model forest tree genus *Populus* [10]. Overall, we found that the microbial communities of soil and plant compartments of *R. stylosa* had both unique and shared features with the other model plants. Shared features included: (i) rhizospheres have the highest bacterial and fungal richness [10,70], (ii) the endospheric bacterial community is frequently lower in leaves than in other plant organs [10,73], and (iii) the fungal communities in root and aboveground plant compartments are generally dominated by Ascomycota [10,68]. The unique features detected in *R. stylosa* compared to other model plants were as follows. First, fungi highly dominated the endosphere microbiome of all plant organs. In other model plants, both bacteria and fungi have been robustly detected in endospheres of plant organs, while the plants we examined were extreme in that we did not detect bacteria in endospheres of all their organs. Second, composition patterns of bacterial and fungal communities detected in the *R. stylosa* rhizosphere compartment differed from those of model plants reported in previous studies [10,69,70,71,72]. Generally, Proteobacteria, Acidobacteria, Actinobacteria form the backbone of the bacterial rhizosphere microbiomes in the model plants. However, we rarely detected Acidobacteria in the rhizosphere of *R. stylosa* (relative abundance ~4%). We also frequently detected Planctomycetes and Chloroflexi (relative abundance >10%). In some specific soil types and locations, these two bacterial phyla also reportedly co-dominate the rhizosphere of *Oryza* spp. [70,71]. Third, Dikarya (Ascomycota and Basidiomycota) were the only fungi detected in the rhizosphere and endospheres (root, stem and leaf) of *R. stylosa*, while other, lower fungi, such as Zygomycota and Chytridiomycota, etc. were apparently absent. In compartments of other model plants, both Dikarya and lower fungi have been detected. In *Populus* spp. and *Oryza* spp., lower fungi of the Zygomycota even co-dominate the rhizosphere and/or root (in *Populus* spp.) microbiomes [10,71]. Locations, soil types, and environmental factors also strongly affect the taxonomic composition of bacterial and fungal communities at coarse and fine taxonomic resolutions in these model plants [69,70,72,73,74]. Thus, similar microbiome studies of *R. stylosa* should be conducted in other locations with different environmental conditions to confirm if the unique pattern observed in our study is specific for this environment or is a general pattern for this tree species.

## Figures and Tables

**Table 1:** Goodness-of-fit statistics (*R*^2^) for factors fitted to the non-metric multidimensional scaling (NMDS) ordination of fungal community composition of indicated plant compartments.

Variables	NMDS1	NMDS2	*R* ^2^	*p*
N	−0.24449	−0.96965	0.3017	0.118
C	0.91384	0.40609	0.7252	0.002
C/N	0.61409	0.78924	0.7074	0.002
Na	−0.76628	−0.64251	0.3764	0.049
K	−0.03703	0.99931	0.1363	0.465
Ca	0.80126	0.59831	0.5309	0.017
Mg	−0.99599	0.08946	0.2532	0.176

**Table 2:** Results of analysis of similarities (ANOSIM) and nonparametric multivariate analysis of variance (NPMANOVA) based on Jaccard distances and presence/absence data in comparisons of fungal community composition in indicated pairs of soil and plant compartments.

Comparison	ANOSIM	NPMANOVA
	*R*	*F*
Leaf vs. root	0.64 *	2.79 *
Leaf vs. rhizosphere soil	1.00 *	5.37 *
Leaf vs. stem	0.84 *	2.96 *
Root vs. rhizosphere soil	0.79 *	2.93 *
Root vs. stem	0.79 *	2.66 *
Rhizosphere soil vs. stem	1.00 *	4.09 *

* *p* < 0.05.

## Supplementary Materials

The following are available online at https://www.mdpi.com/2076-2607/7/12/585/s1.
