# Supplementary material for: First Insights into the Microbiome of a Mangrove Tree Reveal Significant Differences in Taxonomic and Functional Composition among Plant and Soil Compartments
Source: Microorganisms. 2019 Nov 20;7(12):585. doi: 10.3390/microorganisms7120585 (PMC6955992; doi:10.3390/microorganisms7120585)
Supplement: Supplementary file 1 [file microorganisms-07-00585-s001.zip › microorganisms-609193-Supplementary Materials/Microorganism-609193-Supplementary.pdf]

Supplementary Materials

First insights into the microbiome of a mangrove tree reveal significant differences in taxonomic and functional composition among plant and soil compartments

Potential author names and contributions:

Witoon Purahong[1,*,¶], Dolaya Sadubsarn[1,2,*], Benjawan Tanunchai[1,*], Sara Fareed Mohamed Wahdan[1,3], Chakriya Sansupa[1,4,5], Matthias Noll[6], Yu-Ting Wu[7,¶], François Buscot[1,8]

Affiliations:

[1] UFZ-Helmholtz Centre for Environmental Research, Department of Soil Ecology, 06120 Halle (Saale), Germany
[2] Furtwangen University, Department of Medical Life and Science, Faculty Bio and Process engineering, 78054 VS-Schwenningen, Germany
[3] Department of Botany, Faculty of Science, Suez Canal University, 41522 Ismailia, Egypt
[4] Biology department, Faculty of Science, Chiang Mai University, 50200 Chiang Mai, Thailand
[5] Graduate School, Chiang Mai University, 50200 Chiang Mai, Thailand
[6] Coburg University of Applied Sciences and Arts, Institute for Bioanalysis, 96450 Coburg, Germany
[7] National Pingtung University of Science and Technology, Department of Forestry, 91201 Pingtung, Taiwan
[8] German Centre for Integrative Biodiversity Research (iDiv), 04103 Leipzig, Germany

* These authors contributed equally to this work.

¶ Correspondence: witoon.purahong@ufz.de (W.P.); yutingwu@mail.npust.edu.tw (Y.-T.W.)

Figure S1. Rarefaction curves of plant and soil-plant compartments across all samples.

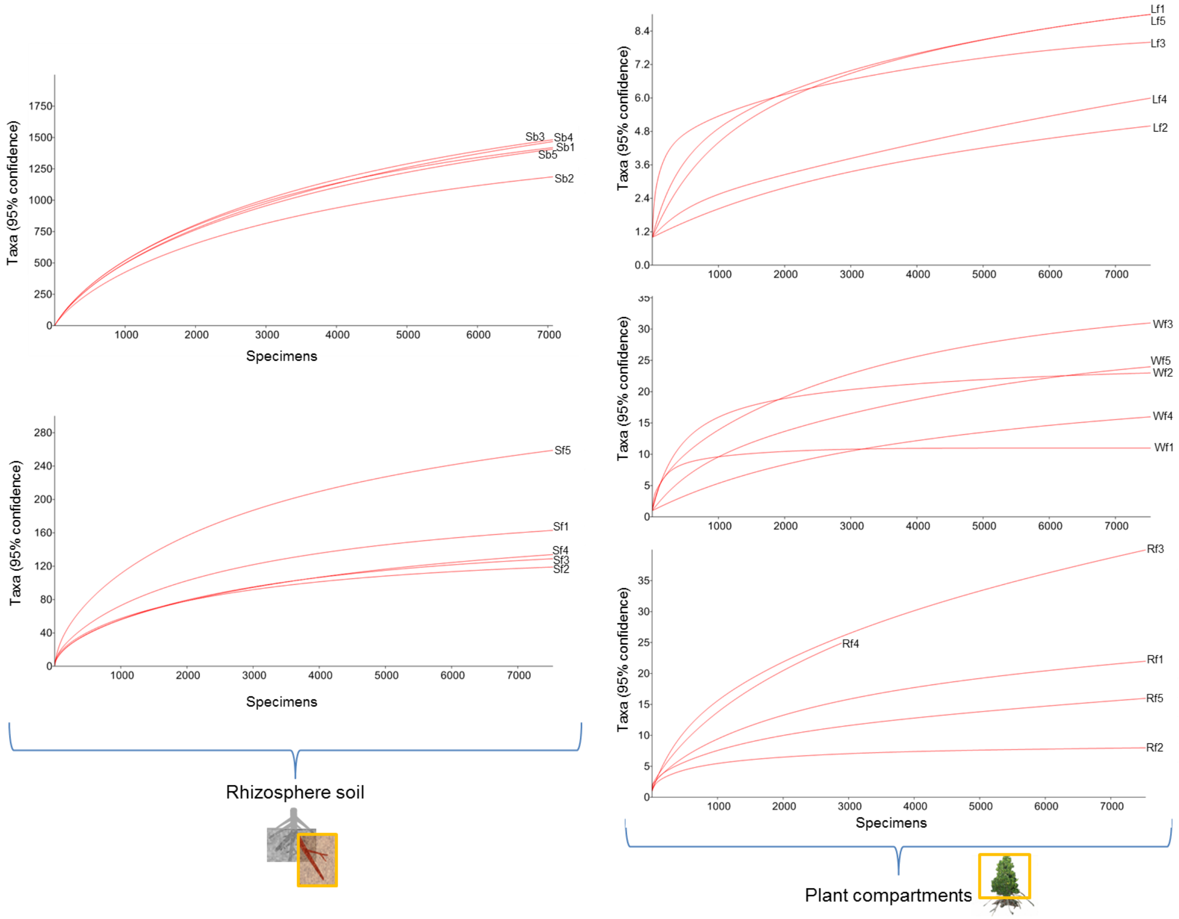

Figure S2. PCR products of bacteria using Bac341F/907v and Bac341F/ Bac785R primers (used in this study).

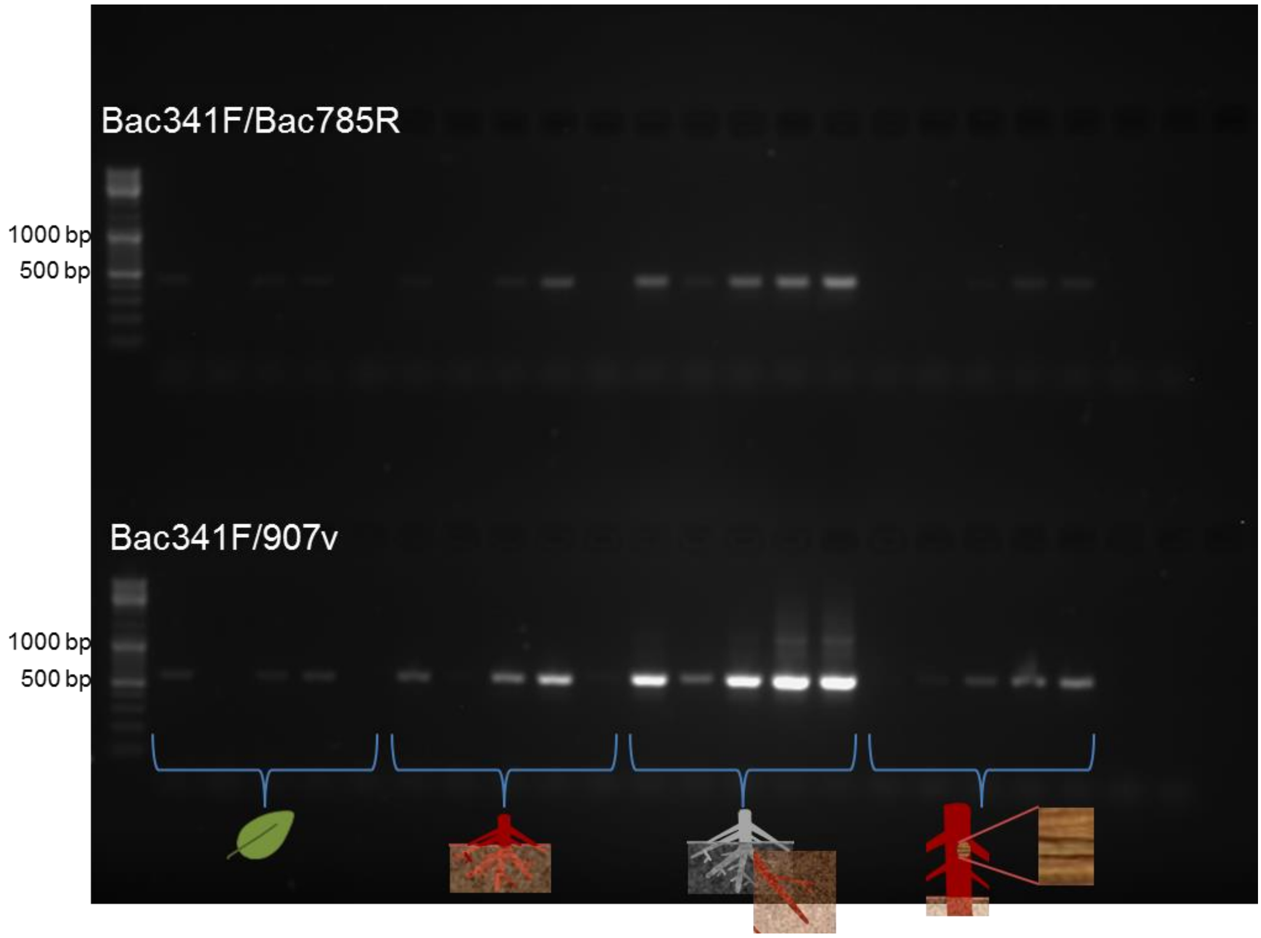

Figure S3 Ecological functional groups assigned to 396/2,497 bacterial OTUs detected in this study.

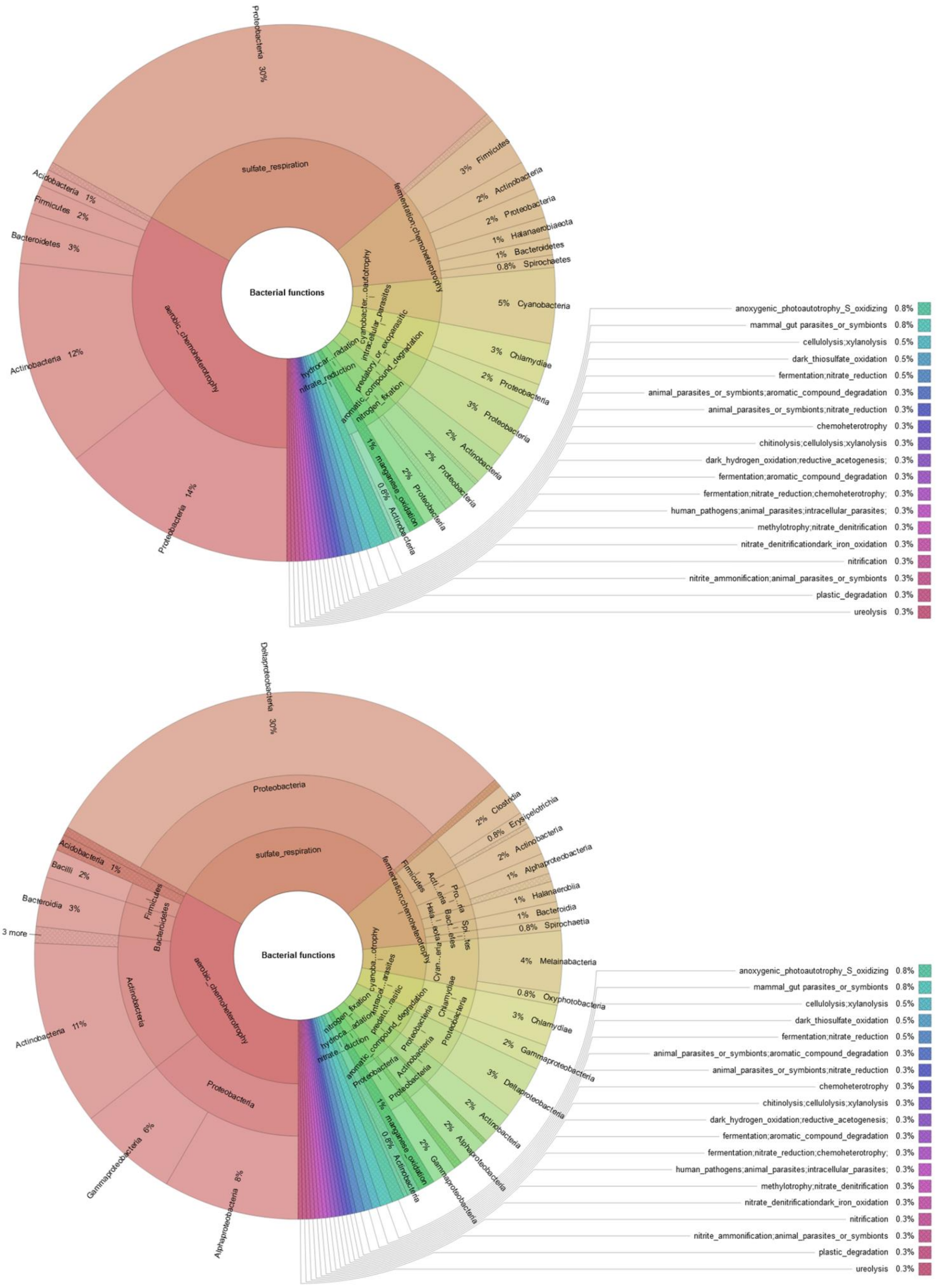

Figure S3 Ecological functional groups assigned to 396/2,497 bacterial OTUs detected in this study (continue).

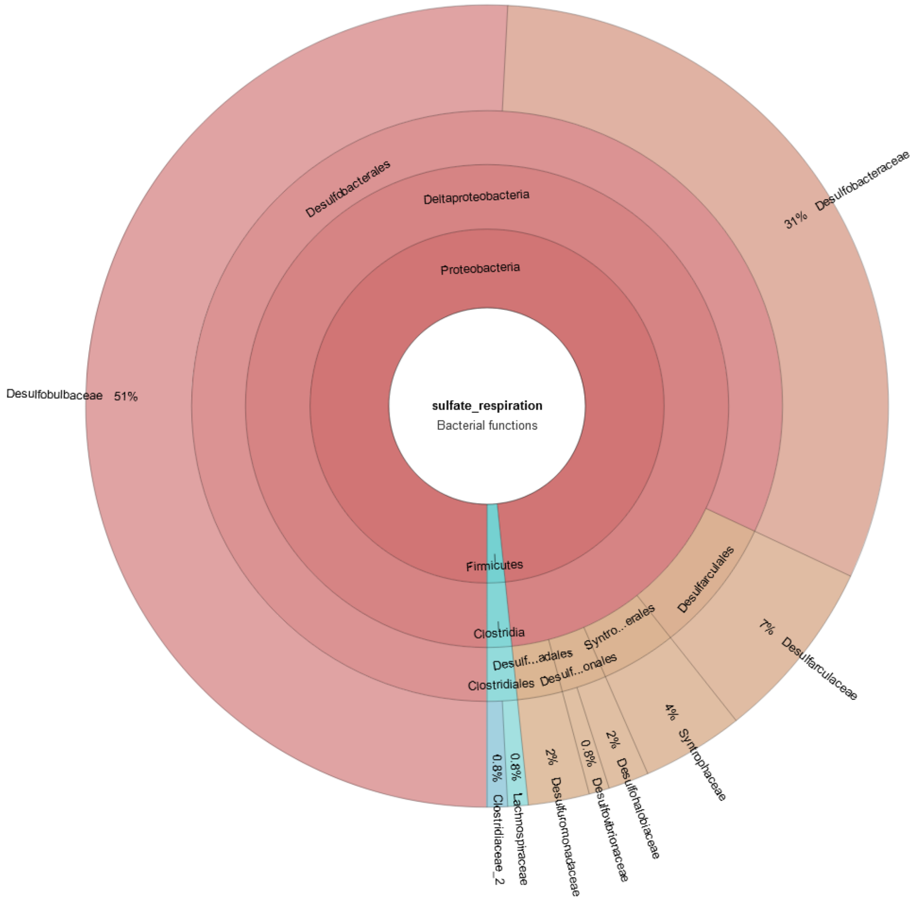

Zoom in: sulfate respiration function

Figure S4 Schematic drawing how fungi may be transported from rhizosphere soil through xylem and eventually reach leaf and other aboveround organs.

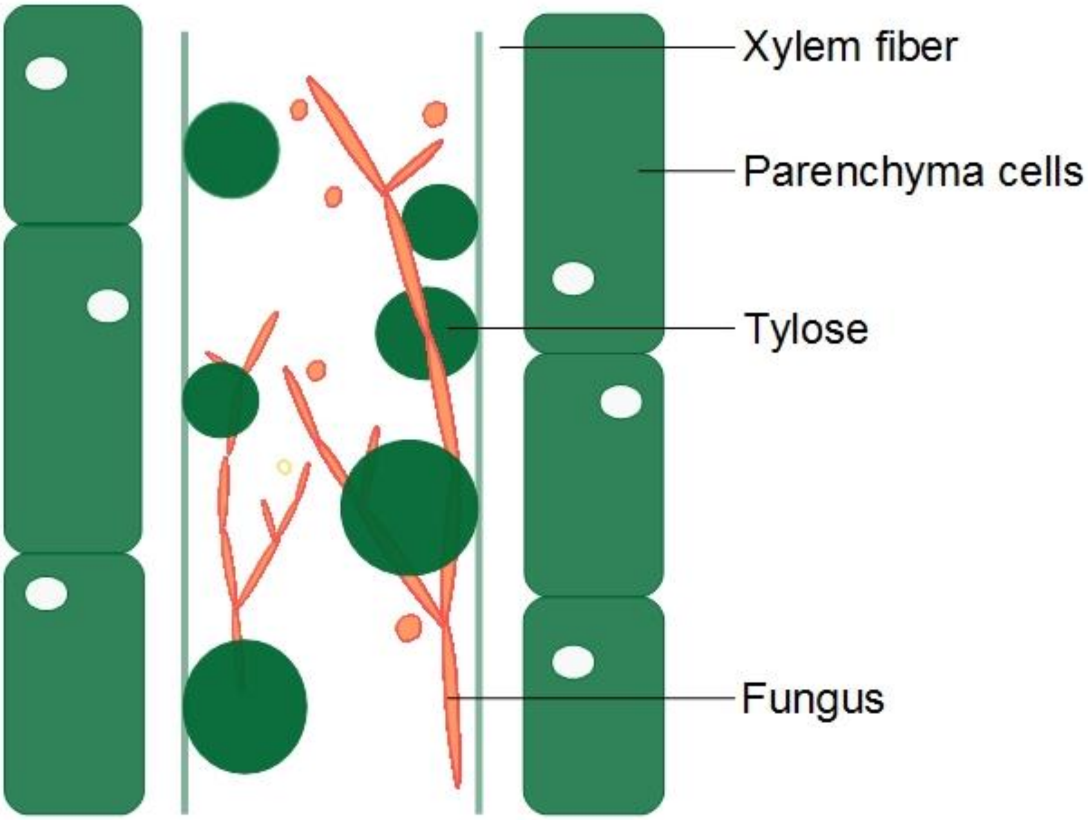

Table S1 Information on bacterial community compositions at fine taxonomic resolution and ecological functions based on FAPROTAX. Some nitrogen fixing bacteria were identified based on Velázquez et al., 2017.

Velázquez, E.; García-Fraile, P.; Ramírez-Bahena, M.-H.; Rivas, R.; Martínez-Molina, E. Current status of the taxonomy of bacteria able to establish nitrogen-fixing legume symbiosis. In Microbes for legume improvement; Springer International Publishing: Cham, Germany, 2017; pp. 1–43.

Please see Excel Table S1.

Table S1.1 Information on all detected bacterial OTUs.

Table S1.2 Information on all functionally assigned bacterial OTUs

Table S2 Goodness-of-fit statistics (R^2) for factors fitted to the non-metric multidimensional scaling (NMDS) ordination of bacterial community composition in rhizosphere soil.

Variables	NMDS1	NMDS2	R^2	*p*
N	-0.12	0.99	0.68	0.292
C	0.99	0.10	0.87	0.183
C:N	0.89	-0.45	0.98	*0.092*
pH	0.03	0.99	0.67	0.31
EC	0.55	-0.83	0.80	0.18

Table S3 Nutrients in different soil-plant compartments with mean ± SE (n = 5).

Variables	Root (mean ± SE)	Stem (mean ± SE)	Leaf (mean ± SE)	Rhizosphere soil (mean ± SE)
N	0.68 ± 0.04	0.32 ± 0.02	1.17 ± 0.05	0.10 ± 0.15
C	27.00 ± 3.63	52.29 ± 0.21	49.91 ± 0.64	0.71 ± 0.15
C:N	39.79 ± 4.37	166.32 ± 10.88	43.18 ± 2.13	7.47 ± 1.91
Na	87.77 ± 29.27	13.26 ± 0.05	8.11 ± 0.48	ND
K	8.22 ± 4.31	6.33 ± 0.27	6.39 ± 0.16	ND
Ca	4.49 ± 2.74	11.77 ± 0.21	12.62 ± 0.39	ND
Mg	13.23 ± 1.14	9.19 ± 0.40	11.88 ± 0.50	ND

Table S4 Goodness-of-fit statistics (R^2) for factors fitted to the non-metric multidimensional scaling (NMDS) ordination of fungal community composition in different soil-plant compartments.

Variables	NMDS1	NMDS2	R^2	*p*
N	-0.29	0.96	0.13	0.338
C	-0.91	-0.41	0.81	**0.001**
C:N	-0.45	-0.89	0.58	**0.002**

Table S5 Similarity percentage (SIMPER) of bacteria in rhizosphere soil samples (Rs3 *vs.* Rs5) based on Bray-Curtis distance with overall average dissimilarity = 49.27

Taxon	Av. dissim	Contrib. %	Cumulative %	Mean abund. Rs3	Mean abund. Rs5
Actinomarinales OTU00002	1.214	2.463	2.463	382	210
Caldilineaceae OTU00010	0.9879	2.005	4.469	38	178
Propionibacteriaceae OTU00009	0.9738	1.977	6.445	43	181
Chromatiaceae OTU00032	0.6351	1.289	7.734	8	98
Chloroflexi OTU00008	0.5574	1.131	8.866	47	126
Ilumatobacter OTU00003	0.4939	1.003	9.868	230	160
Halochromatium OTU00067	0.374	0.7591	10.63	5	58
Thermomicrobiales OTU00015	0.3599	0.7304	11.36	91	40
Sandaracinaceae OTU00022	0.3599	0.7304	12.09	43	94
Caldilineaceae OTU00064	0.3528	0.7161	12.8	5	55

Table S6 Similarity percentage (SIMPER) of fungi in rhizosphere soil samples (Rs3 *vs.* Rs5) based on Bray-Curtis distance with overall average dissimilarity = 39.86

Taxon	Contrib. %	Cumulative %	Mean abund. Rs3	Mean abund. Rs5
Aspergillus niger OTU0005	7.685	36.81	88	550
Penicillium steckii OTU0015	5.007	47.09	3	304
Teratosphaeria sp OTU0013	3.16	54.14	25	215
Setophaeosphaeria hemerocallidis OTU0018	2.462	56.6	0	148
Pleosporales_unclassified OTU0007	2.329	58.93	118	258
Aspergillus flocculosus OTU0021	2.096	63.19	1	127
Penicillium coffeae OTU0012	1.996	65.19	31	151
Penicillium citrinum OTU0014	1.68	68.86	16	117
Monosporascus sp. OTU0006	1.514	72.02	2	93
Agaricomycetes_unclassified OTU0026	1.331	73.35	0	80
